# “More work means less pay”: a qualitative understanding of physicians’ patient selection decision-making under DRG reform in Nanjing, China

**DOI:** 10.1186/s12939-026-03044-1

**Published:** 2026-09-21

**Authors:** Xiaoying Zhu, Daniel Ll Strachan, Shenglan Tang, Tiara Marthias, Ajay Mahal, Barbara McPake

**Affiliations:** 1 School of Elderly Care Services and Management, Nanjing University of Chinese Medicine, Nanjing, Jiangsu 210023 China; 2 Nossal Institute for Global Health, Melbourne School of Public Health, The University of Melbourne, Melbourne, Victoria 3053 Australia; 3 Global Health Research Center, Duke Kunshan University, Kunshan, Jiangsu 215316 China; 4 Duke Global Health Institute, Duke University, Durham, NC 27710 USA; 5 SingHealth Duke-NUS Global Health Institute, Duke-NUS, Singapore, 169857 Singapore

**Keywords:** Health insurance, Cream skimming, Physician behaviour, Diagnosis-related group, Financial incentive

## Abstract

**Background:**

Patient selection, also known as ‘cream-skimming’, can lead to the systematic exclusion of high-cost, clinically complex patients from necessary care. It has been identified as an opportunistic response under Diagnosis-Related Groups (DRGs) payment systems globally. As China advances the rollout of DRG reform alongside commitments to promoting healthcare equity, understanding the ecology of physicians’ cream-skimming behaviour and how to mitigate it is imperative.

**Methods:**

We purposively sampled 21 physicians from two hospitals with different incentive structures in 2024 and conducted semi-structured face-to-face interviews on their experiences with and responses to DRG reform. Data were analysed using an inductive thematic approach followed by deductive framework analysis.

**Results:**

We found that, in this study setting, physicians often assessed whether the expected reimbursement would cover the anticipated treatment costs when making admission decisions, with patient selection emerging as an adaptive response to DRG payment incentives. Their decisions were shaped by interacting factors related to capability, opportunity, and motivation. Capability captured physicians’ confidence in managing financially risky cases; opportunity encompassed the physical and social conditions that enabled them to balance financial risk with quality of care; and motivation concerned the value they placed on different outcomes. Physicians were more likely to admit financially risky patients when three dimensions aligned: they believed that these patients could be effectively managed under DRG payment, expected their decisions to produce meaningful and recognised outcomes, and valued those outcomes, including patient welfare and the fulfilment of professional obligations.

**Conclusions:**

The findings suggest that physicians’ responses to DRG payment are driven not only by formal payment rules but also by how financial risks are perceived and mediated within hospitals and the broader health system. Improving equitable access requires realigning performance metrics with clinical needs, strengthening referral and post-acute care pathways, and reducing reliance on individual risk-based decision-making through team-based support.

**Supplementary Information:**

The online version contains supplementary material available at https://doi.org/10.1186/s12939-026-03044-1.

## Introduction

Prospective hospital reimbursement arrangements based on Diagnosis-Related Groups (DRGs) payment method have proliferated worldwide, aiming to improve service efficiency and to contain hospital expenditure through reshaping incentive structures [1]. Under DRG-based reimbursement design, cases classified within a particular DRG are characterised by similar clinical diagnoses and broadly homogeneous patterns of resource use, and hospitals receive a fixed rate for each admission depending on a patient’s diagnosis [2]. Therefore, hospital revenue per case does not fully reflect the resources used in treating individual patients, and they are theoretically incentivised to limit unnecessary costs to receive increased compensation for their services. However, the new incentive structure might also encourage opportunistic practices that affect hospital performance and the effectiveness of the payment reform, as documented under DRG payment internationally [3–5]. Among these unintended practices, patient selection, also known as ‘cream-skimming’ that reduces the number of admitted patients with higher anticipated cost profiles has been viewed as a main reason for the failure of prospective payment systems to improve hospital performance [6].

The introduction of prospective payment can be regarded as one of the most significant structural reforms in China’s health financing system. By the end of 2024, China’s social health insurance system covered approximately 1.33 billion people [7], making it the largest social health insurance system worldwide. At the same time, hospital expenditure in China has continued to rise, roughly doubling in real terms between 2013 and 2023, reaching 5,582 billion yuan [8]. In pursuing universal health coverage, the Chinese government has faced mounting cost pressure on health insurance funds driven by rapidly rising hospital expenditure and has consequently advanced a series of provider payment reforms intended to improve health system efficiency since 2009. Among these reforms, DRG payment has become an important alternative to traditional fee-for-service (FFS) reimbursement. By the end of 2023, it had been implemented in 190 health insurance pooling areas [9], accounting for roughly half of all pooling areas nationwide.

In principle, DRG payment may improve access by creating incentives to increase the number of admissions, while encouraging providers to limit treatment intensity within each admission. This is because payment is primarily determined at the case level, whereas the marginal revenue from providing additional services within an admission is generally zero [10]. However, given the reliance on bundled payments and average-cost pricing underlying the DRG-based payment design, providers may be discouraged from admitting patients whose expected costs fall above the “average” case underlying the cost model [11]. This practice arises from the coexistence of two fundamental objectives within hospitals: financial sustainability and the quality and safety of care. While these objectives can mutually reinforce each other, sometimes they are in conflict [12]. Excluding complex or high-risk patients may therefore be a way to resolve this conflict. In China, facing these embedded financial incentives for patient selection under DRG payment reform, the government has issued policy commitments aimed at preventing the refusal or diversion of patients on the basis of DRG payment standards [13]. Achieving the policy objectives relies on the admission decisions made by frontline physicians. However, little is known about how physicians navigate these competing pressures in their daily admission decisions.

Some researchers in China have examined the potential emergence of patient selection, indicating that health providers selectively admitted patients with lower-than-average costs within a DRG to reap financial gains [14, 15], or shifted complex cases to the conventional FFS payment system to reduce financial risks where DRG coverage was partial at the early stages of implementation [16]. The findings are particularly relevant in tertiary hospital settings. A systematic review documented that DRG gaming behaviours in China, including patient selection and cost-shifting, were predominantly reported in tertiary hospital settings [16]; Zhou et al. [17] found that tertiary hospitals exhibited significantly higher rates of DRG gaming behaviours than secondary hospitals. The deviations between the DRG grouping scheme and actual clinical practice, as well as inadequate regulatory oversight, are commonly emphasised as key factors contributing to the patient selection behaviour in these studies. However, it is likely physicians’ admission decisions under cost-containment environments are shaped by multiple, interacting factors operating at different levels [18]. Yet empirical evidence remains scarce. Moreover, much of the existing evidence is derived from analyses based on hospital-level data, with limited examination of the decision-making mechanisms of individual physicians. Nanjing, the capital of Jiangsu Province in eastern China, provides a relevant setting in which to examine physicians’ admission decision-making under DRG payment reform. Nanjing has a high concentration of tertiary healthcare resources and receives patients from both within and outside the city. As part of China’s nationwide reform of inpatient payment, the city formally established a DRG payment framework in 2021 [19], initially covering secondary and tertiary medical institutions, with full coverage of all designated medical institutions providing inpatient services implemented by 2023 [20].

This study adopts a qualitative approach to (1) identify factors shaping physicians’ responses to the financial incentives embedded in DRG payment in their admission decisions; (2) explain the mechanisms through which these factors interact to influence physicians’ admission decisions under DRG payment reform.

## Methods

### Study design

A qualitative design using semi-structured interviews was employed in our study. We drew upon the COM-B model of behaviour as the primary analytical framework to systematically identify capability (C), opportunity (O), and motivation (M) factors influencing physicians’ admission decisions (B) when responding to the financial incentives embedded in DRG payment. Developed by Michie et al. [21], the COM-B model proposes that for individuals to engage in a behaviour, they require physical and psychological capability, appropriate social and physical opportunity, and motivation, either through automatic processes or reflective mechanisms. It has been widely used in the fields of individual health behaviours and clinical practice [22, 23].

**Fig. 1:**
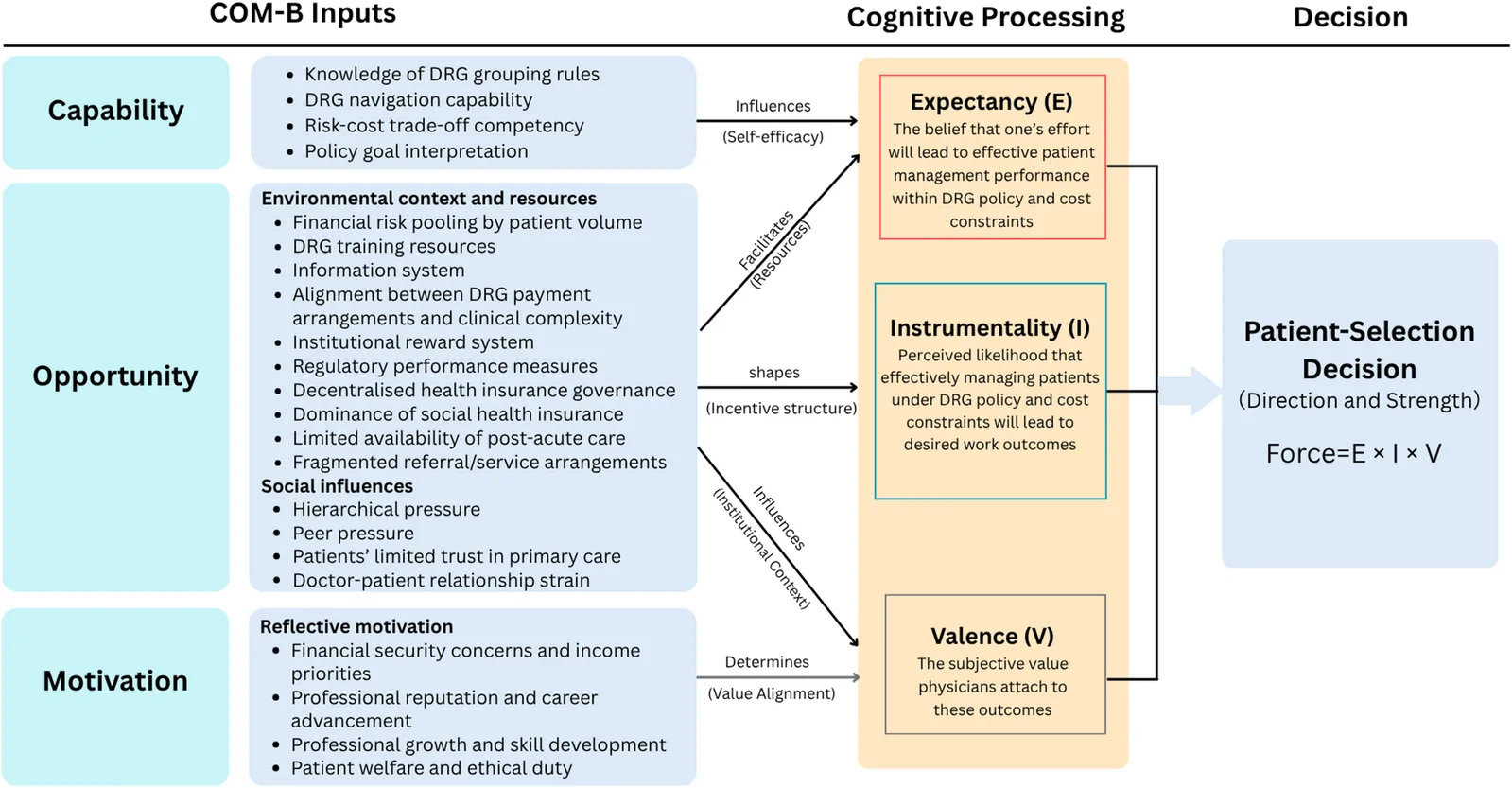
Mechanisms of physicians’ patient-selection behaviour across COM-B components

The COM-B model was selected because its determinants-oriented focus aligned with the study aim of identifying the behavioural influences shaping physicians’ admission decisions. In addition, it provides a comprehensive yet parsimonious structure for organising and presenting the range of individual and organisational influences identified in the data [21]. However, it does not explain in detail how individuals assess alternative courses of action when influences across different COM-B domains compete. Therefore, we drew on Expectancy Theory [24] to unpack the cognitive mechanisms. The theory provided a complementary interpretive lens to explain how physicians evaluated the capability-, opportunity-, and motivation-related influences identified through COM-B when choosing between competing behavioural pathways (see Fig. 1 in the Discussion section). This study forms part of a broader qualitative research programme examining health providers’ experiences of China’s DRG payment reform. While previous analyses explored a broad range of physicians’ responses to the payment [25], this paper focuses specifically on how physicians make patient selection decisions and the implications of these decisions for equitable access to hospital care.

### Participants, recruitment and data collection

We purposefully focus on tertiary hospitals as information-rich settings, as existing evidence suggests that DRG gaming behaviours, including patient selection, are more commonly reported in tertiary than in secondary hospitals in China [16, 17]. Their heterogeneous case mix under DRG payment may intensify incentives for selective admission [16]. Participants were recruited from two public tertiary hospitals in Nanjing, Jiangsu Province in China. Within China’s policy and technical framework, Nanjing adopted a points-based DRG payment method operating under a city-wide global budget. Each DRG was assigned a base number of points, calculated primarily using historical cost data, and the monetary value of each point was pre-set for monthly pre-settlement and then dynamically finalised at the end of the fiscal year by dividing the total regional budget by the aggregate points generated. Hospitals were financially accountable for differences between DRG-based reimbursement and treatment costs. The policy included adjustment mechanisms for atypical cases. During the study period, low-cost outliers, defined as cases with total inpatient costs below 40% of the city-wide mean cost for the corresponding DRG, were reimbursed based on actual expenditure. For high-cost critically ill cases, cases are initially reimbursed according to the standard DRG settlement arrangements, leading to a temporary financial gap for the hospital. A proportion of the highest-ranked cases within each hospital subsequently underwent retrospective medical-record review by the municipal health insurance authority. For approved cases, the reasonable excess costs were compensated during the annual final settlement [19, 20].

To deter undesirable patient selection behaviour, Nanjing Healthcare Security Administration has explicitly linked them to healthcare insurance reimbursement, whereby settlement points are deducted in proportion to the frequency and severity of the violation [26]. The two hospitals were purposefully selected because their internal incentive arrangements differed from each other, providing variation in organisational contexts for analysis. The selection was information-driven, with findings from interviews in the first hospital informing the inclusion of a second hospital with a different internal incentive arrangement. One hospital linked staff performance income to DRG surpluses, whereas the other emphasised departmental management rather than directly linking income and surpluses. Data collection continued within both hospitals until no substantively new information relevant to the study aim emerged. Additional hospital sites were therefore not sought, as the two settings provided sufficient contextual variation and the interviews generated sufficient depth to examine the underlying behavioural mechanisms.

Purposive sampling was used to recruit participants who met the following eligibility criteria: being a hospital-based physician, working in inpatient care, and having at least five years of clinical experience, thereby enabling them to provide more informative accounts of the impact of DRG introduction. Participant recruitment aimed to achieve diversity in medical specialities, career stages, and gender. We used a snowball sampling method to identify suitable participants. Upon initial contact, potential participants were informed about the nature and purpose of the study, assured of confidentiality, the expected duration of the interview, and their rights as participants, including their right to decline any questions they felt uncomfortable with and to withdraw from the interview at any time. A plain language statement was provided prior to the interview.

Data collection took place in May and June 2024. We recruited participants until data saturation was achieved [27]. Data were generated through one-on-one, in-person interviews. To protect participants’ confidentiality and minimise interruptions, interviews were conducted in quiet offices within hospital wards or in meeting rooms at each hospital. All interviews were undertaken by the first author (XYZ), who has experience in qualitative interviewing in China. Interviews were conducted in Mandarin and audio-recorded with participants’ consent. The data were de-identified at the point of transcription.

### Data analysis

Interviews were transcribed verbatim and imported into NVivo 14 for data management and analysis. The first author (XYZ), a bilingual researcher, transcribed all interviews and translated three complete transcripts into English to facilitate review by the wider research team. For the remaining interviews, relevant excerpts were translated as needed to support codebook development and team discussions. Data were coded thematically using a combined inductive and deductive approach [28]. To preserve context-specific meanings and subtle cultural assumptions, the first author conducted the primary coding using the original-language transcripts, while co-authors contributed to theme refinement, interpretation, and the mapping of themes to the COM-B framework.

The analysis involved two stages. In the first stage, enablers and barriers were identified inductively based on the data itself. In the second stage, these inductively derived themes were deductively mapped onto the COM-B model. The COM-B domains and their corresponding subdomains (physical and psychological capability, physical and social opportunity, and reflective and automatic motivation) provided a pre-defined theoretical framework for organising and interpreting the themes. The initial mapping was iteratively reviewed and refined according to each theme’s dominant behavioural relevance. This process involved repeated movement between data-driven interpretation and theory-informed categorisation. Throughout this process, themes and their alignment with the COM-B framework were discussed with co-authors (BM and DS) to critically examine and refine the interpretations. Themes with unclear alignment to particular COM-B domains were discussed until consensus was reached. To enhance the rigour and transparency of the analytic process, the definition of each code, theme refinement, and the rationale for assigning themes to specific COM-B domains were documented through iterative revisions of the codebook.

The first author had more than ten years of experience in health policy research in China, including research on public hospital reform, as well as experience in conducting qualitative interviews in China. While this background supported an in-depth understanding of the policy and organisational contexts in which participants’ accounts were situated, it also had the potential to shape the interpretation of the data. The research team comprised researchers with expertise in international health systems research, health economics, and sociology. Reflexive discussions among team members were used to critically examine emerging interpretations and challenge assumptions arising from experiential perspectives.

### Ethics statement

Ethical approval was obtained from the University of Melbourne (ID: 2024-28945-51804-3). The study protocol was evaluated and deemed exempt from ethical approval by the local institutional ethics committee in China, in compliance with national minimal-risk research guidelines. All participants provided informed consent.

## Results

Twenty-one physicians were purposively sampled (Table 1), including 9 from Hospital X, which implemented direct internal DRG-related economic incentives, and 12 from Hospital Y, which had no such incentive. All participants had over five years of clinical experience. Six also held managerial roles, such as department directors (4) or administrators in the hospital’s health insurance department (2).

**Table 1:** Participant characteristics

Characteristics	Median [IQR], (range) or *n* (%)
Age (years)	39 [37,43], (31–55)
Male	10 (48%)
Year of experience	
6–10 years	5 (24%)
11–15 years	9 (43%)
16–20 years	4 (19%)
20 + years	3 (14%)
Professional title	
Resident Physician	3 (14%)
Attending Physician	3 (14%)
Associate Chief Physician	9 (43%)
Chief Physician	6 (29%)
Affiliation	
Hospital X	9 (43%)
Hospital Y	12 (57%)

IQR: interquartile range; Hospital X, with direct personal financial incentives; Hospital Y, without direct personal financial incentives

Our analysis focuses on the factors influencing physicians’ admission decisions under the incentives created by DRG payment, particularly when treating patients whose expected costs may exceed reimbursement. To better understand these factors, the findings are structured around the three core domains of the COM-B model: (1) Capability; (2) Opportunity; (3) Motivation. There were two domains of the COM-B for which we did not identify any matched data: the ‘Physical capability’ under the ‘Capability’ and ‘Automatic motivation’ under the ‘Motivation’. We elaborate on the most salient themes below, while a comprehensive overview of the barriers and enablers across COM-B dimensions is provided in appendix 1.

### Capability

Participants’ psychological capability was found to influence their admission decisions under DRG payment, particularly when treating patients with high expected costs or clinically complex patients.

#### Knowledge of DRG grouping rules

Participants described limited understanding of DRG grouping rules, particularly the core treatment criteria used to manage regulatory risk, as a key capability barrier influencing their admission decisions. During DRG grouping, cases that did not meet the core treatment requirements of the assigned DRG group were considered non-compliant by the health insurance authority. After repeatedly having such admissions flagged as “non-compliant” and without ever being clear about the reasons, participants tended to avoid admitting these cases to reduce the risk of further violations. One physician described this dilemma as follows:

*For some cases*,* the core treatment criteria are quite opaque……I’ve submitted a lot of appeal materials*,* but none of them worked.* (Hospital Y, Resident Physician, P21)

#### DRG navigation capability

Participants reported uncertainty around estimating and managing treatment costs for complex or severe cases, which made it difficult to assess potential financial risk when making admission decisions under DRG payment. Conversely, adequate DRG navigation capability was perceived as an enabler for admitting those cases. Participants with such capability were able to anticipate treatment costs and the likely reimbursement level and demonstrated an understanding of cross-case resource reallocation between low-cost and high-cost patients to achieve overall cost control. They also demonstrated proficiency in applying DRG grouping and pricing standards, enabling them to strategically code to better align reimbursement with actual treatment costs. The navigation capability increased their confidence in managing complex cases within DRG cost constraints.

*For severe cases*,* if the costs are higher than the insurance payment level*,* we don’t necessarily avoid admitting them*,* because cost control is quite volatile. For example*,* some patients recover in just a few days and don’t use up their insurance budget*,* then the remaining budget can help offset costs for other patients*,* as long as the overall costs stay roughly within limits.* (Hospital Y, Associate Chief Physician, P04)

#### Risk-cost trade-off competency

Physicians emphasised the importance of their competency in weighing medical risks against financial constraints. This enabled them to make clinical decisions that balance patient wellbeing with cost-control requirements under DRG payment.

*This physician needs to weigh these considerations: “Well*,* this patient is elderly*,* but health insurance only pays for this case with 20*,*000 yuan. What should I do? I may have to reduce some services.” The potential risks then depend largely on the physician’s level of expertise. It’s best if one can navigate and minimise omissions.* (Hospital Y, Chief Physician, P01)

### Opportunity

Both physical and social opportunities were reported as important in shaping supportive environments that enabled physicians to balance financial risks and quality of care when making admission decisions. Within this domain, environmental context and resources captured material resources and broader organisational, institutional, and health-system conditions that enabled or constrained admission decisions, while social influences captured influences operating through interpersonal relationships, social norms, expectations, and pressures [21, 29].

### Environmental context and resources

#### Financial risk pooling by patient volume

Some physicians, particularly those from hospital X, where DRG surpluses were linked to physicians’ income, emphasised that patient volume affected the capacity of hospitals or departments to pool financial risks. Specifically, small bed capacity constrained their willingness to admit patients with uncertain costs or lengths of stay, leading them to prioritise more predictable cases. At the same time, the large patient volume was considered to enable departments to pool financial risks across cases and offset the inadequate reimbursement with high-cost cases under DRG payment. The large patient volume was reflected in not only greater bed capacity but also substantial outpatient visits and a high proportion of cross-regional patients.

*Health providers can disregard DRG constraints only when the inpatient care can be subsidised through a high volume of outpatients… They just need to make sure the inpatient costs are reasonable and don’t go too far over the DRG limit.* (Hospital X, Chief Physician, Department Head, P16)

#### Alignment between DRG payment arrangements and clinical complexity

Physicians reported that treatment costs could vary substantially across patients within the same DRG group. They felt that some DRG groupings did not fully capture differences in clinical complexity. As a result, reimbursement was sometimes insufficient to cover the costs of treating patients with greater clinical complexity, while milder cases in the same group generated financial surpluses. This tended to create differential financial incentives in admission decisions.

*Take kidney stones as an example*,* they fall under a code called LC19. This code covers a range of procedures*,* including both rigid and flexible ureteroscopy. But the costs for flexible and rigid scopes are actually quite different*,* so lumping them together under the same code isn’t really reasonable.* (Hospital Y, Resident Physician, P07)

Although one participant mentioned that some cases involving cost overruns might receive additional compensation from the health insurance authority through a special-case review mechanism at the end of the year, this was not identified by other participants as a factor in their admission decision-making.

#### Limited availability of post-acute care

Beyond factors directly related to the DRG policy, broader health system-level factors, particularly limited post-acute care, were found as barriers for physicians to admit older or severely ill patients. Participants reported that, due to the cost-containment requirements, patients’ length of stay is restricted, making it impossible for physicians to provide extended inpatient care as they did previously. At the same time, limited options for post-discharge care could make it difficult to transition some patients out of acute hospitals. These difficulties could also operate through social influences by contributing to strains in doctor-patient relationships, which in turn made some physicians more cautious about admitting such cases.

*This is not only about the problem of DRG but also about the problem of the whole health system…… A lot of older patients stay in hospital for a long time. There’s nowhere suitable for them to go*,* so they’re forced to keep transferring between hospitals. However*,* the health insurance policy doesn’t permit repeated inter-hospital transfers*,* so it also creates a lot of trouble for us. We can only try our best to avoid the risks that may come to our department.* (Hospital X, Associate Chief Physician, P13)

#### Institutional reward system

Physicians described the importance of an institutional reward system that protects them when admitting high-cost patients. One physician from the hospital which linked the DRG surplus to the physician’s income described that *‘The current situation is that when it comes to the treatment of critically ill patients*,* more work means less pay*,* which is the most direct consequence.’* (Hospital X, Chief Physician, Department Head, P17).

Such direct financial incentive structures embedded within hospital or departmental reward systems were perceived to encourage profit-oriented behaviours and to influence physicians’ clinical decision-making at the first step of the care pathway. In contrast, participants from the hospital that did not link the DRG-surplus to physician’s income indicated that institutional financial protection enabled them to maintain their clinical focus. This protection was identified as operating at two levels of revenue allocation: from hospitals to departments and from departments to individual physicians.

*Our hospital is actually protecting departments financially*,* shielding them from losses.* (Hospital Y, Resident Physician, Administrator, P20)

*In our department*,* we basically still calculate performance as a group*,* because we’ve considered that if the incentive is fully implemented*,* no one would be willing to take on the critically ill or complex cases.* (Hospital X, Chief Physician, Department Head, P11)

These different institutional reward arrangements appeared to affect the extent to which the financial consequences of DRG performance were passed on to individual physicians and, consequently, the salience of financial considerations in their admission decisions.

#### Regulatory performance measures

Physicians noted that they are accountable not only to DRG-related assessment indicators from health insurance authorities but also to institution or department performance evaluations conducted by health administrative authorities. Regulatory performance measures, particularly those embedded in the National Tertiary Public Hospital Performance Appraisal, such as the case-mix index (CMI), reflecting service capacity, and patient satisfaction indicators, reflecting service quality, were reported as important considerations in physicians’ decisions to admit complex patients in line with their clinical needs.

*We are now also paying more attention to admitting some seriously ill patients*,* such as those who hope to be admitted in our department and who we are able to treat…Otherwise our CMI value will be very low.* (Hospital X, Associate Chief Physician, P14)

To avoid dissatisfaction, participants noted that they often admit patients even when they anticipate the patient might be a high-cost case.

*A patient with multiple organ failure*,* every organ needs support*,* which obviously costs a lot more. But if you don’t admit them to the hospital*,* they’ll file a complaint against you.* (Hospital Y, Associate Chief Physician, P19)

#### Decentralised health insurance governance

Participants reported that due to the localised nature of health insurance governance, hospitals’ cost control performance for non-local patients is not evaluated by local insurance authorities. This influenced them to prioritise the admission of these patients as a way to avoid DRG cost constraints.

*Patients with local health insurance aren’t always as advantageous as those with non-local insurance or self-paying patients*,* so that can affect our priorities at the admission stage.* (Hospital Y, Associate Chief Physician, P09)

#### Fragmented referral and service arrangement

Participants described weak referral coordination and poor continuity of care across different levels of the health systems as limiting their ability to manage patients efficiently.

*In my view*,* if you want to follow the tiered healthcare model in other countries*,* you need to build the foundation first. Why could they implement it successfully? Because they had proper referral systems*,* public awareness*,* and everything in place. Here*,* we just talk about saving money and reducing the burden.* (Hospital Y, Associate Chief Physician, P19).

Although physicians sometimes arranged transfers, these typically relied on informal personal networks rather than institutionalised processes. For financially risky cases, some physicians were hesitant to make such informal referrals to avoid transferring risk.

*Sometimes I do help them with a referral*,* because some patients are almost like your friends… But some patients make you feel they could turn against you at any moment. For those cases*,* I don’t want to bring trouble to my colleagues or friends.* (Hospital X, Associate Chief Physician, P18).

### Social influences

#### Hierarchical pressure

Even without direct financial incentives, the hierarchical pressure within the hospital was reported by physicians as an important factor influencing their clinical decisions. They were expected to consider both DRG policy compliance and institutional cost-containment goals, based on directives issued at the institutional level or communicated through departmental leadership.

*Currently*,* there’s no implementation of such personal income deductions in our hospital. However*,* during weekly meetings*,* the hospital director puts pressure on the department heads*,* who then pass that pressure down to us.* (Hospital Y, Resident Physician, P07)

#### Peer pressure

Participants reported that although their DRG-related financial performance was assessed at the department rather than individual level, their performance was regularly summarised and made visible within the department. This meant that colleagues were aware of who generated substantial DRG losses, creating the potential for peer pressure.

*If I admit this patient*,* it might bring down our team’s overall financial performance. If I keep doing this*,* it will definitely affect my colleagues’ interests.* (Hospital X, Associate Chief Physician, P15)

#### Patients’ limited trust in primary care

Participants observed that many patients prefer to seek care directly at high-level hospitals for their initial visit, even when their conditions are relatively mild and could be managed at lower-level facilities. Participants attributed this partly to patients’ limited trust in primary care services, which constrained the role of primary care facilities in managing less complex patients and limited their capacity to reduce patients’ tendency to seek initial care at tertiary hospitals.

*In fact*,* current policies show a clear tendency to promote the tiered healthcare system. For example*,* registration fees are much lower at primary care institutions*,* and medication costs are about 10% cheaper at secondary hospitals than at tertiary hospitals. But the problem is that*,* in general*,* Chinese patients still don’t trust the lower-level institutions.* (Hospital X, Chief Physician, Department Head, P16)

### Motivation

Our findings indicated that physicians’ admission decisions were shaped by reflective motivation [30], comprising four key aspects. These motivations often co-existed within individual physicians, requiring them to balance different outcomes in their decision-making.

**Financial security concerns and income priorities** were perceived as barriers to avoiding patient selection. Participants from hospital X consistently expressed the view that admitting complex or high-cost patients may threaten departmental financial sustainability or their own personal income security under DRG payment reform. To avoid financial loss, participants perceived the selective behaviour as a way of meeting various performance indicators.

**Professional reputation and career advancement** functioned both as barriers to and enablers of physicians refraining from patient selection. On one hand, physicians described being more cautious about admitting severely ill patients when cost-containment pressures made it difficult to meet patients’ expectations around hospital stay duration, particularly given concerns about potential complaints and their implications for professional reputation. On the other hand, performance indicators linked to professional title promotion were reported as encouraging more inclusive admission practices among physicians.

*Doctors often see their professional title as a sign of achievement*,* since it reflects both their research capacity and their clinical level.* (Hospital Y, Chief Physician, P01)

**Professional growth and skill development** emerged as an enabler for admitting complex cases. Participants described the sense of professional satisfaction and fulfilment they gained through their clinical practice, particularly when successfully managing challenging cases or receiving patient recognition. They believed that their medical expertise could be enhanced through treating complex cases, whereas prolonged admission of only mild cases would not diminish their clinical competencies.

**Patient welfare and ethical duty** emerged as an important enabler for participants’ non-selective behaviours. This could be reflected by physicians’ ethical reflections on the broader consequences of DRG payment policies, specifically whether such policies may compromise equitable access to care. As one participant described:

*Healthcare is not just about a payment issue or a cost issue. If you want to reduce costs*,* it’s actually very simple – you can choose not to treat many cases … From a kind of ‘God’s-eye view’, patients like this (with a terminal illness) are seen as just consuming social resources. So*,* the question becomes: should we give up on them? What these policies do is abandon these patients.* (Hospital Y, Associate Chief Physician, P19).

Some physicians described the dilemma they faced, as one noted that ‘*The most stressful part of this situation for me is that*,* with my intelligence*,* I can do the math about money and figure out the benefits*,* but doing so takes away the sincerity of what I’m doing*.’ (Hospital X, Associate Chief Physician, P15).

## Discussion

Under the dual incentives created by the DRG payment, providers are encouraged to increase patient volume because each additional eligible admission can generate additional reimbursement, while they also face financial risks when treating patients whose expected costs exceed reimbursement. This creates a tension in physicians’ admission decisions. Drawing on evidence from the two hospitals studied, we identified factors shaping physicians’ admission decisions in response to these incentives embedded in DRG payment, using the COM-B model as an analytical framework. Critically, the analysis reveals that physicians’ responses to DRG payment are shaped not only by the payment rules themselves but also by how financial risks and incentives are perceived and mediated within hospitals and the broader health system. This finding is consistent with international studies highlighting the relevance of organisational conditions and managerial emphasis on financial performance to patient-selection incentives and responses [31–33]. Our findings extend this literature by identifying how broader health-system conditions, alongside these organisational and managerial factors, shape physicians’ capacity and willingness to admit financially risky patients under DRG payment.

While the COM-B model allowed us to categorise the identified determinants of physicians’ responses, the patterns emerging from the findings invite a deeper examination of the cognitive mechanisms through which these components translate into physician behavioural decision-making. The reflective decision-making process identified among physicians in this study appears to align with Vroom’s Expectancy Theory, an influential theory in behavioural decision-making [24]. It posits that individuals choose between behavioural options by evaluating anticipated outcomes and the perceived likelihood of achieving them, based on three key components: *expectancy*, *instrumentality*, and *valence*. A person is motivated to engage in a specific behaviour when they believe that (1) effort will lead to acceptable performance (expectancy), (2) performance will lead to valued outcomes (instrumentality), and (3) these rewards are desirable (valence). We have drawn on Expectancy Theory to assist interpretation of how the behavioural determinants identified through the COM-B analysis may interact in physicians’ admission decision-making.

Consistent with theoretical research showing that prospective payment can influence treatment choices through the relationship between reimbursement and costs [5], our findings suggest that such financial considerations may arise even earlier, when physicians decide whether to admit high-cost or clinically complex patients. In this study setting, physicians often evaluated whether the expected reimbursement would be sufficient to cover the anticipated treatment costs when making admission decisions for high-cost or clinically complex cases. This evaluation created a perceived trade-off between clinical effort and financial risk. From the perspective of expectancy theory, physicians form expectations about whether additional effort will allow them to manage high-cost or clinically complex patients within reimbursement constraints. Differences in these expectations may partly explain variation in their responses. Some physicians demonstrated low expectancy and, as a result, attempted to mitigate potential financial losses using patient selection as one strategy among a small number. In our study, physicians’ expectations appeared to be shaped by their perceived capability to balance patient wellbeing with cost-control requirements and their perceived ability to navigate the DRG system. Notably, the awareness of cross-case resource reallocation across patients emerged as a key enabler.

We observed that physicians’ admission decisions were also influenced by factors related to the environmental context and resources, as well as social influences. Among these factors, regulatory performance measures and institutional reward systems were found to be particularly prominent. Drawing on expectancy theory, these factors shaped physicians’ perceptions of instrumentality, which refers to the perceived link between successfully managing financially risky patients under DRG constraints and valued outcomes. In China, public hospitals and physicians’ performance are subject to monitoring by healthcare security authorities as well as performance evaluations conducted by health authorities. These regulatory measures aim to ensure the quality and appropriateness of healthcare provision. We found that physicians’ admission decisions reflect a balance between financial risks and their anticipated departments’ performance evaluation results. For instance, admitting complex cases may improve case-mix index (CMI) evaluations, an indicator of hospitals’ clinical capacity. However, at the level of individual cases, certain complex cases may require resources beyond what physicians consider feasible under DRG payment. Such cases may therefore carry a higher risk of patient dissatisfaction or complaints. Patients perceived as more demanding or difficult to manage may further increase the likelihood of complaints, leading physicians to anticipate potential negative evaluation results.

In addition to external regulatory oversight, internal reward system design was regarded as playing an important role in shaping the perceived relationship between performance and its potential consequences. In our study, the hospital linking DRG surpluses to physicians’ income transferred the financial risks to individual physicians, leading them to perceive that admitting patients whose anticipated treatment costs might exceed DRG reimbursement would result in personal financial loss. This perception strengthened incentives to avoid such cases. By contrast, in the hospital without direct income incentives, hierarchical pressure functions as an alternative source of instrumentality, implicitly conveying expectations regarding both cost control and clinical quality requirements. Given that hospital administrators exert substantial influence over physicians’ career advancement in China [17], physicians may anticipate career-related consequences of their decisions. These findings suggest that multiple incentives operate simultaneously in shaping physicians’ admission decisions. When DRG-related financial incentives are not fully transmitted to individual physicians, hospitals may create a combination of incentives that moderates pure cost-containment pressures while maintaining incentives for clinical performance. Such incentive bundling may help mitigate tensions between financial considerations and service quality in physicians’ decision-making. However, as Zhou et al. [17] noted, hospitals’ responses to DRG incentives also depend on their cost-management capacity, which requires enabling support from relevant government authorities, including technical guidance from the National Healthcare Security Administration.

Our analysis suggests that some features of the broader health system may shape how physicians perceive the consequences of their admission decisions. We found that the decentralized governance in healthcare insurance created opportunities for patient selection that can bypass DRG constraints, for example, prioritising non-local patients who were perceived as financially less risky because they were not subject to the same local DRG payment arrangements. During the study period, patients insured outside the Nanjing municipal pooling area could access reimbursement through cross-regional medical insurance settlement [34], but their cases were not formally included in Nanjing’s local DRG payment and associated performance assessment. Participants therefore perceived these patients as financially less risky, because their treatment costs were not constrained by the same fixed DRG payment applied to locally insured patients. In addition, the long-standing tendency in China for patients to seek initial care directly at high-level hospitals, including those with relatively mild conditions [35], exposes physicians to a heterogeneous pool of patients and expands their discretion in admission decisions. Further, this pattern reflects perceived limitations in the capacity of primary care services [36] and the absence of structured referral pathways. In the absence of such pathways, physicians may have greater scope to justify non-admission on clinical grounds, potentially facilitating cream-skimming and the effective prioritisation of patients with lower levels of clinical need. The limited availability of post-acute care represents another health system level problem. This particularly affects clinically complex patients or elderly patients requiring complex acute care. When making admission decisions, some physicians in our study considered these patients may face discharge barriers and remain in hospital for longer periods, which would reduce bed turnover efficiency. As a result, these internal hospital efficiency pressures and external system constraints create a context in which admitting high-need complex patients may be perceived as incompatible with other desired outcomes.

The motivational factors identified in our study revealed that physicians simultaneously valued multiple outcomes, which were at times conflicting. This motivational tension and decision-making process could be explained by the ‘Valence’ dimension of Vroom’s Expectancy Theory, which denotes the subjective value individuals place on those outcomes [24]. In our study, physicians were found to assign positive valence to outcomes related to patient welfare and ethical duty, as well as to professional growth and skill competence, while simultaneously placing high value on financial security. Although valence is grounded in individual values, evidence from healthcare workers in Malawi and employees in the United States and Germany suggests that the value attached to work-related outcomes may also be shaped by cultural and organisational contexts, including workplace culture, recognition, and working conditions [37, 38]. Consistent with this evidence, our findings suggest that institutional and organisational factors tended to influence how physicians weighed and prioritised competing outcomes. We established Fig. 1 to provide a summary of the key themes identified in this study and show how they intersect with the COM-B components and Expectancy Theory to inform physicians’ decision-making.

The core principle of expectancy theory emphasises the multiplicative relationship among its three components [24]. This implies that under the DRG financial incentives, physicians are more likely to admit financially risky patients when all three components (E, I, V) are present, namely when they believe that these patients can be successfully managed, anticipate meaningful consequences from admission decisions, and attach value to outcomes such as patient welfare and professional responsibility. Our findings suggest that these components are interrelated and shaped by contextual factors, including institutional reward arrangements, which can influence how strongly DRG-related financial consequences are transmitted to individual physicians and how these consequences are weighed alongside patient welfare and professional obligations. This can, in turn, amplify or attenuate their combined effect on equitable admission decisions.

## Contributions and implications for practice

This study contributes to the literature on physicians’ behavioural responses to DRG payment [16, 25] by identifying the mechanisms through which DRG incentives are consciously processed by physicians when they make admission decisions. While economic incentives inherent in DRG systems tend to encourage cream-skimming, empirical findings vary across studies [15, 16, 39]. Our findings help explain this variation by showing that physicians’ admission decisions are shaped through a more complex motivational process, in which their expectations of case management, perceptions of organisational consequences, and the value they attach to different outcomes combine to influence decision-making. Our findings highlight the importance of strengthening the behavioural and organisational conditions that support physicians’ motivation to promote equitable access to health services. These include redesigning performance metrics to avoid penalising complexity among presenting patients, improving referral and post-acute care availability, and enhancing team-based support that reduces physicians’ reliance on individual risk assessments.

In practice, the monitoring of health providers’ cream-skimming largely relies on complexity-based indices derived from administrative data. However, potential unintended behaviours, such as upcoding (refers to assigning codes to higher case-mix classifications for higher reimbursement) [17, 25] and context based factors, such as variations in the proportion of non-local patients across hospitals, may distort these measures. Our qualitative study complements these quantitative analyses by illuminating physicians’ behavioural dynamics and the underlying contextual factors. In addition, while the COM-B model of behaviour was originally intended to provide a common language that can be connected and compared with other theories for deeper theoretical exploration [30], most existing applications in healthcare settings have applied the model in isolation. Our study offered a practical example of integrating the COM-B model with other psychological theories to better understand individual behaviour in the context where these behaviours take place.

Several limitations should be considered when interpreting the findings. First, this study was based on a purposively selected sample of physicians from two public tertiary hospitals in Nanjing, China. The findings may therefore not be transferable to secondary or primary care facilities operating with different resource and case-mix profiles. Moreover, although DRG payment in China follows a nationally guided overarching framework, its specific design and implementation vary considerably across pooling areas. Caution is therefore required when transferring the specific findings of this study to other regions operating under different prospective payment arrangements. Nevertheless, the study may offer transferable insights into how physicians assess financial and clinical risks under prospective payment, particularly in settings with similar payment arrangements. Further studies exploring whether themes generated in this analysis are present across contexts are recommended. In addition, because this study interviewed physicians only, it captures physicians’ perceptions and understanding of DRG policy and organisational arrangements. Although these perceptions are directly relevant to understanding their admission decisions, physicians’ accounts cannot provide a complete assessment of formal policy design or its implementation. Future studies incorporating policymakers and hospital managers could offer complementary insights and examine potential gaps between formal policy design and frontline interpretation.

## Supplementary Information

Below is the link to the electronic supplementary material.

Supplementary Material 1

## Data Availability

The data are not publicly available because they contain information that could compromise participant anonymity. Relevant excerpts are provided in the manuscript. Requests for access to confidential data will be considered on a case-by-case basis for researchers who meet the relevant criteria. Data enquiries can be directed to the corresponding author.
