# Supplementary material for: “More work means less pay”: a qualitative understanding of physicians’ patient selection decision-making under DRG reform in Nanjing, China
Source: Int J Equity Health. 2026 Sep 21;25:220. doi: 10.1186/s12939-026-03044-1 (PMC13602623; doi:10.1186/s12939-026-03044-1)
Supplement: Supplementary file 1 [file 12939_2026_3044_MOESM1_ESM.docx]

**Supplementary materials**

|  |  | Barriers | Enablers |
| --- | --- | --- | --- |
| Capability | Psychological capability | Limited knowledge of DRG grouping rules | Adequate DRG navigation capability |
|  |  | Cost prediction deficiency in complex cases | Risk-cost trade-off competency |
|  |  | Disconnect in understanding policy goals |  |
| Opportunity | Environmental context and resources | Small inpatient capacity limiting financial risk pooling | Large patient volume enabling financial risk pooling |
|  |  | Misalignment between DRG grouping rules and clinical complexity | Functional information system |
|  |  |  | Available DRG training resources |
|  |  | Limited availability of post-acute care | Special-case review mechanism |
|  |  | Institutional reward system reinforcing profit-oriented decision-making | Departmental financial risk sharing |
|  |  |  | Institutional financial protection enabling clinical focus |
|  |  | Decentralized governance in healthcare insurance | Dominance of social health insurance |
|  |  | Fragmented health system | Regulatory performance measures |
|  | Social influence | Hierarchical pressure |  |
|  |  | Peer pressure |  |
|  |  | Patients’ limited trust in primary care |  |
|  |  | Doctor-patient relationship strain |  |
| Motivation | Reflective motivation | Financial security concerns and income priorities | Patient welfare and ethical duty |
|  |  | Professional reputation and career advancement | Professional growth and skill development |
|  |  |  | Professional reputation and career advancement |

**Table 1. Factors shaping physicians’ responses to DRG incentives in admission decisions**
